# Weak self-association of cytochrome *c* peroxidase molecules observed by paramagnetic NMR

**DOI:** 10.1007/s10858-016-0035-z

**Published:** 2016-05-28

**Authors:** Jesika Schilder, Marcellus Ubbink

**Affiliations:** Gorlaeus Laboratories, Leiden Institute of Chemistry, Leiden University, Einsteinweg 55, 2333 CC Leiden, The Netherlands

**Keywords:** Cytochrome *c*, Ultra-weak interactions, Paramagnetic relaxation enhancement, Ensemble docking, Electron transfer

## Abstract

**Abstract:**

There is growing experimental evidence that many proteins exhibit a tendency for (ultra)weak homo- or hetero- oligomerization interactions. With the development of paramagnetic relaxation enhancement NMR spectroscopy it has become possible to characterize weak complexes experimentally and even detect complexes with affinities in the 1–25 mM range. We present evidence for a weak complex between cytochrome *c* peroxidase (CcP) molecules. In a previous study, we attached nitroxide based spin labels at three positions on CcP with the intent of observing intramolecular PRE effects. However, several intermolecular PRE effects were also observed suggesting a weak self-association between CcP molecules. The CcP–CcP complex was characterized using paramagnetic NMR and protein docking. The interaction occurs between the surface that is also part of the stereo-specific binding site for its physiological partner, cytochrome *c* (*Cc*), and several small, positively charged patches on the “back” of CcP. The CcP–CcP complex is not a stereo-specific complex. It is a dynamic ensemble of orientations, characteristic of an encounter state. The contact areas resemble those observed for CcP molecules in crystals. The CcP–CcP complex formation competes with that of the CcP-Cc complex. However, the affinity for Cc is much larger and thus it is expected that, under physiological conditions, auto-inhibition will be limited.

**Graphical Abstract:**

A weak self-association between cytochrome *c* peroxidase molecules was characterized using paramagnetic NMR.
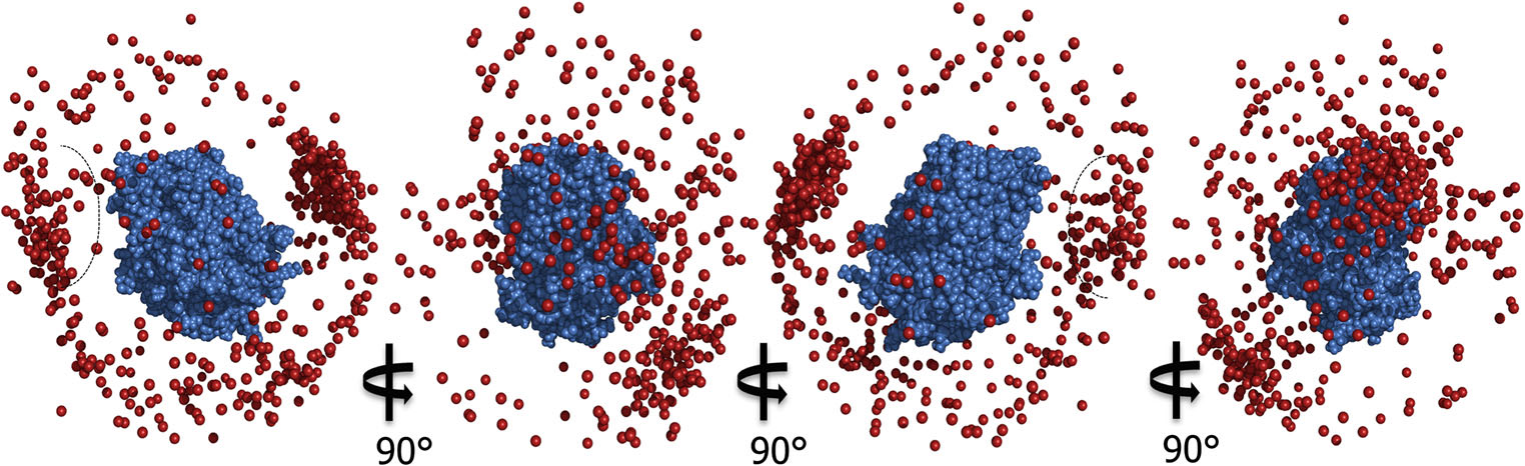

**Electronic supplementary material:**

The online version of this article (doi:10.1007/s10858-016-0035-z) contains supplementary material, which is available to authorized users.

## Introduction

Decades of research on protein–protein interactions have revealed valuable information about the structure and function of many protein complexes. While the majority of this work has focused on proteins that form strong and often highly specific interactions, the importance of the complexes of weakly associated proteins is becoming increasingly clear. These transient complexes are commonly formed to counter-balance the biological need for a specific interaction with the necessity of maintaining a high turnover rate (Schilder and Ubbink 2013). Transient complexes also include ultra-weak interactions, defined as having a dissociation constant (*K*_D_) in the millimolar range, (Tang et al. 2008a, b; Vaynberg and Qin 2006) that are known to drive self-assembly of higher order homogeneous architectures like crystals, viral capsids and amyloid fibrils (Fawzi et al. 2007; Garcia-Ruiz 2003; Zlotnick 2005). They also play an important role in an array of cellular processes including rapid assembly/disassembly, protein maturation, reversible cell adhesion and cell signalling (Vaynberg and Qin 2006). While strongly associated protein complexes often consist of low energy, specific states that are easily isolated and studied, weakly associated protein complexes often also occupy higher energy conformations, such as the encounter state (Kleckner and Foster 2011; Ubbink 2009). These conformations are lowly populated, transient and cannot be isolated, making them practically invisible to conventional structural biology techniques (Clore 2011).

The development of new paramagnetic nuclear magnetic resonance spectroscopy (NMR) techniques has enabled characterization of these transient states in populations as low as 0.5 % (Keizers and Ubbink 2011). Paramagnetic relaxation enhancement (PRE) is particularly well-suited to studying lowly populated states, as the dipolar interaction with the unpaired electron is very strong and the strength of the PRE falls off with the sixth power of the distance between the paramagnetic centre and observed nucleus, making PRE extremely distance dependent (Iwahara and Clore 2006; Tang et al. 2006; Volkov et al. 2006). PRE has been successfully used to characterize several encounter complexes (Fawzi et al. 2010; Hiruma et al. 2013; Scanu et al. 2012; Suh et al. 2007; Volkov et al. 2006; Xu et al. 2008, 2009) including the cytochrome *c* (Cc)-cytochrome *c* peroxidase (CcP) encounter complex (Bashir et al. 2010; Schilder et al. 2014; Van de Water et al. 2014; Volkov et al. 2006, 2010a). It has also been used to study protein-DNA complexes (Iwahara and Clore 2006; Iwahara et al. 2004, 2006) as well as large scale domain motions (Henzler-Wildman et al. 2007; Tang et al. 2007) and transient structures in unfolded and intrinsically disordered proteins (Bertoncini et al. 2005; Dedmon et al. 2005; Gillespie and Shortle 1997a, b; Shortle and Ackerman 2001).

In 2008, PRE was used for the first time to visualize ultra-weak self-association (*K*_D_ ≥ 15 mM) between histidine-containing phosphocarrier protein (HPr) molecules as these dimers could not be observed by other techniques. Paramagnetic EDTA-Mn^2+^ tags were placed at three positions on the surface of unlabelled HPr. This protein was mixed 1:1 (300 μM each) with [^15^N]-labelled HPr and PRE were measured. An ultra-weak self-association was observed, representing a population of 1 %, which disappeared when the physiological HPr binding partner, enzyme I, was added. Furthermore, this interaction could be modulated by changes in the ionic strength or with charge mutations (Tang et al. 2008a). In the same year, PRE was used to show an ultra-weak interaction between the N-terminal extension of the HIV-1 protease precursor and the protein’s active site resulting in autocleavage and maturation of the protein. The ultra-weak encounter complex (*K*_D_ = 3–6 mM) was found to represent 3–5 % of the total population in a concentration of 200 μM (Tang et al. 2008b). This technique has since been applied to several ultra-weak complexes (Johansson et al. 2014; Liu et al. 2012; Villareal et al. 2011) with *K*_D_ values as high as 25 mM (Xing et al. 2014). Together, these studies demonstrated the use of PRE for the observing weak interactions of self-associations that are difficult to visualize with conventional methods.

In our previous work using nitroxide based spin labels to obtain intramolecular PRE data on CcP, we observed multiple unexpected PRE effects for residues further than 24 Å from the spin label, the PRE limit for nitroxide radicals (Keizers and Ubbink 2011). Here, we show that these additional PRE are in fact intermolecular effects generated by a weak self-association between the CcP molecules. Furthermore, we have characterized this weak CcP–CcP complex using paramagnetic NMR and ensemble docking. Until now, CcP had been assumed to exist as a monomer in solution. This is interesting for understanding the aggregation propensity of proteins, particularly in the crowded cellular environment. However, the potential biological relevance of a CcP–CcP complex is unclear as the affinity of CcP for Cc is much greater than that for CcP and thus auto-inhibition is unlikely under physiological conditions.

## Materials and methods

### Protein sample preparation

Yeast [^15^N,^2^H]- or [^15^N,^13^C]-labelled CcP C128A with MSKT as the first four N-terminal residues was expressed and purified as published previously (Morar et al. 1999; Pollock et al. 1998; Schilder et al. 2014). The same CcP construct with the additional mutations N38C, N200C or T288C were used to produce unlabelled protein (Schilder et al. 2015; Volkov et al. 2006). 1-acetoxy-2,2,5,5-tetramethyl-δ3-pyrroline-3-methyl)-methanethiosulfonate (MTS) and 1-oxyl-2,2,5,5-tetramethyl-2,5-dihydropyrrol-3-ylmethyl methanethiosulfonate (MTSL) tags were obtained from Toronto Research Chemicals (Toronto, ON, Canada). The spin labels were stored as 100 mM stocks dissolved in DMSO at 4 °C prior to use. The CcP mutants were tagged with MTS, MTSL as described previously (Schilder et al. 2014; Volkov et al. 2006). The tagging efficiency was determined by mass spectroscopy to be essentially 100 %. Yeast iso-1-Cc WT was expressed and purified according to published procedures (Morar et al. 1999; Pollock et al. 1998).

### NMR spectroscopy

All NMR samples contained 20 mM NaPi, 100 mM NaCl, 6 % D_2_O, pH 6.0. The pH of the samples was adjusted to 6.00 ± 0.05, with small aliquots of 0.5 M HCl or NaOH. To determine the optimal CcP concentration, 2D [^15^N,^1^H] TROSY-HSQC (Pervushin et al. 1997) spectra were obtained with 1024 and 128 complex points in the direct and indirect dimensions, respectively, on 400–800 μM double labelled [^15^N,^13^C] CcP samples at 293 K. Measurements were performed at ^1^H Larmor frequencies of 600 MHz on a Bruker Avance III spectrometer equipped with a TCI-Z-GRAD CryoProbe (Bruker, Karlsruhe, Germany). The data were processed using Topspin 3.1 (Bruker, Karlsruhe, Germany).

For inter-molecular PRE measurements, NMR samples contained 200 μM [^15^N,^2^H] labelled CcP WT and 200 μM unlabelled N38C, N200C or T288C CcP with either MTS or MTSL tags attached. For intra-molecular PRE measurements, NMR samples contained 400 μM of [^15^N,^2^H]-labelled tagged mutants. For intra-molecular PRE measurements in the presence of Cc, 400 μM unlabelled WT Cc was also present. 2D BEST-TROSY-HSQC experiments (Lescop et al. 2007) were recorded on a Bruker AVIII HD spectrometer equipped with a ^1^H[^13^C/^15^N] TCI-cryoprobe operating at a proton Larmor frequency of 850 MHz at 293 K with 1024 and 100 complex points in the ^1^H and ^15^N dimensions, respectively. The data were processed using Topspin 3.2 (Bruker, Karlsruhe, Germany). All NMR data were analyzed using CCPN Analysis 2.1.5 (Vranken et al. 2005). The backbone resonance assignment for CcP were taken from (Schilder et al. 2014).

### PRE analysis

The intensity ratio of the amide resonances in the spectra of the paramagnetic (MTSL) and diamagnetic (MTS) samples (*I*_para_/*I*_dia_) was calculated and normalized as described previously (Bashir et al. 2010). The paramagnetic contribution to the transverse relaxation rate, *R*_2,*para*_, was calculated as reported previously (Bashir et al. 2010; Battiste and Wagner 2000; Schilder et al. 2014). For the amide peaks that disappeared in the paramagnetic spectrum, an upper limit for *I*_para_ was set to two standard deviations of the noise level of the spectrum (Schilder et al. 2014).

The calculated *R*_2,*para*_ values were then converted into distances as described previously (Eq. 1) (Bashir et al. 2010):

1 $$r = \sqrt[6]{{\frac{{f_{bound} }}{{R_{2,para} }}\frac{{\gamma_{H}^{2} g_{{_{e} }}^{2} \beta^{2} \mu_{0}^{2} (S + 1)S}}{{240\pi^{2} }}\left( {4\tau_{c} + \frac{{3\tau_{c} }}{{1 + \omega_{H}^{2} \tau_{c}^{2} }}} \right)}}$$

where r is the distance between the oxygen atom of the spin label nitroxide and a given amide proton, *f*_*bound*_ is the fraction of observed protein sample bound to the paramagnetic protein (estimated at 0.40), γ_H_ is the proton gyromagnetic ratio, g_e_ is the electronic g-factor, β is the Bohr magneton, μ_0_ is the vacuum permeability, S is the spin quantum number for the spin label (½) and ω_H_ is the proton Larmor frequency in rad/s (Battiste and Wagner 2000; Bertini et al. 1996). τ_c_ is the correlation time of the vector connecting the radical and the observed nucleus. The τ_c_ is expected to be dominated by the rotational correlation time of the CcP–CcP complex, which was estimated to be 45 ns (Bernado et al. 2002). The calculated distances were divided into three classes: strongly affected residues for which the peaks had been completely broadened out in the paramagnetic spectrum and only an upper limit could be calculated (class I), affected residues for which the peaks were visible in the paramagnetic spectrum (error margins were set to at least ±3 Å to account for experimental error, class II) and residues that were too far away from the spin label to experience significant PRE, so only a lower limit could be calculated (class III) (Bashir et al. 2010; Schilder et al. 2014). We prefer converting PREs to distances rather than to dock directly with PREs because it makes the relation between PRE, τ_c_ and *f*_*bound*_ explicit. In graphical evaluations of back-calculated data, comparing distances puts the emphasis on the most important class II restraints, whereas plotting PREs emphasizes the less defined class I restraints.

### Ensemble docking

**Fig. 1:**
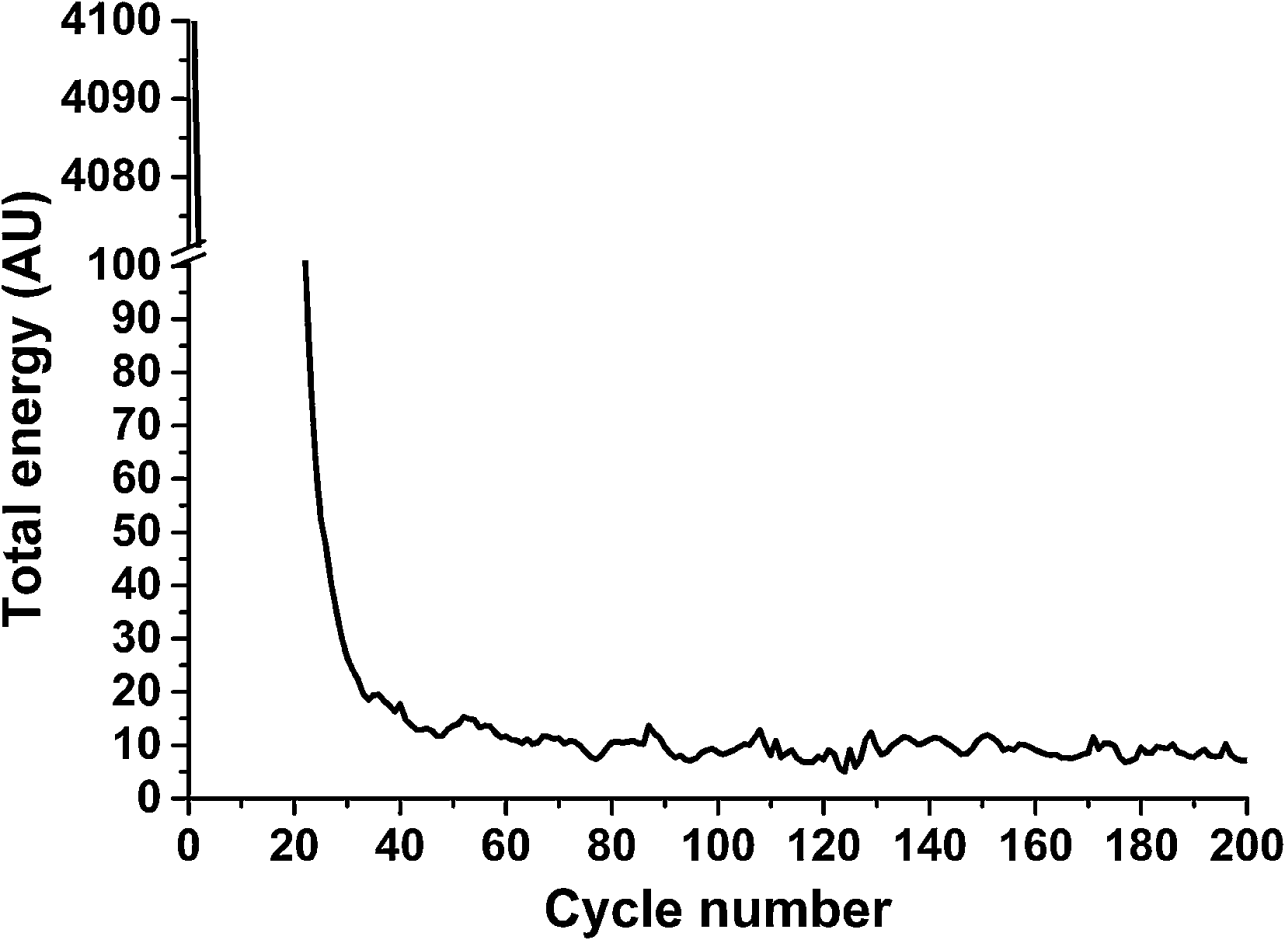
Total energy (in arbitrary units) during a single approach of five copies of CcP with spin label tags on a single untagged CcP. An approach consists of 200 cycles with the lowest energy structure being saved

The coordinates for CcP were obtained from the crystal structure of the complex with Cc, PDB 2PCC (Pelletier and Kraut 1992). The docking of CcP to CcP was driven by a set of distance restraints derived from inter-molecular PRE data using Xplor-NIH version 2.34 (Schwieters et al. 2003, 2006). This was done using an ensemble of four spin label conformers, the orientations of which were fixed in experimentally determined orientations published previously (Schilder et al. 2015). One to eight copies of CcP with spin label tags were docked to untagged CcP using rigid body dynamics with van der Waals repel forces and the distance restraints contributing to the total energy. The distance between the haem iron atoms of the CcP molecules was restrained to 20–60 Å. Docking was repeated from random starting positions using 100 approaches of 200 cycles each (Fig. 1) in which the lowest energy structure of each approach was saved, resulting in 100 structures. One cycle consisted of 1000 steps of 0.4 ps in the dynamics mode of Xplor-NIH.

The twenty lowest energy structures of CcP in the ensemble were analyzed to determine the optimal number of copies of CcP required. With more than five copies of unlabelled CcP in the ensemble, the quality of the fit to the PRE does not improve. Then the docking was repeated for 1000 approaches of 200 cycles each, resulting in 1000 structures of which the 100 lowest energy structures were used to build the ensemble. The back-calculated distances were obtained by taking the r^−6^ average over the four spin label rotamers at each position followed by a linear averaging of the values for the 20 or 100 lowest energy ensemble solutions. The fit between the observed (dis^obs^) and back-calculated (dis^calc^) distances for the class II restraints was evaluated using a Q-factor according to (Eq. 2):(Bashir et al. 2010)

2 $$Q = \sqrt {\frac{{\sum\limits_{i} {\left( {dis_{i}^{obs} - dis_{i}^{calc} } \right)}^{2} }}{{\sum\limits_{i} {\left( {dis_{i}^{obs} + dis_{i}^{calc} } \right)}^{2} }}}$$

Note that in this definition, the denominator is the sum of the observed and calculated distances. The average violation (AV) was determined as described previously, (Schilder et al. 2015) by averaging the difference between the experimental and back-calculated distances; for distances with only an upper (class I) or lower boundary (class III), back-calculated distances that fell inside of those boundaries were not considered violations.

## Results and discussion

**Fig. 2:**
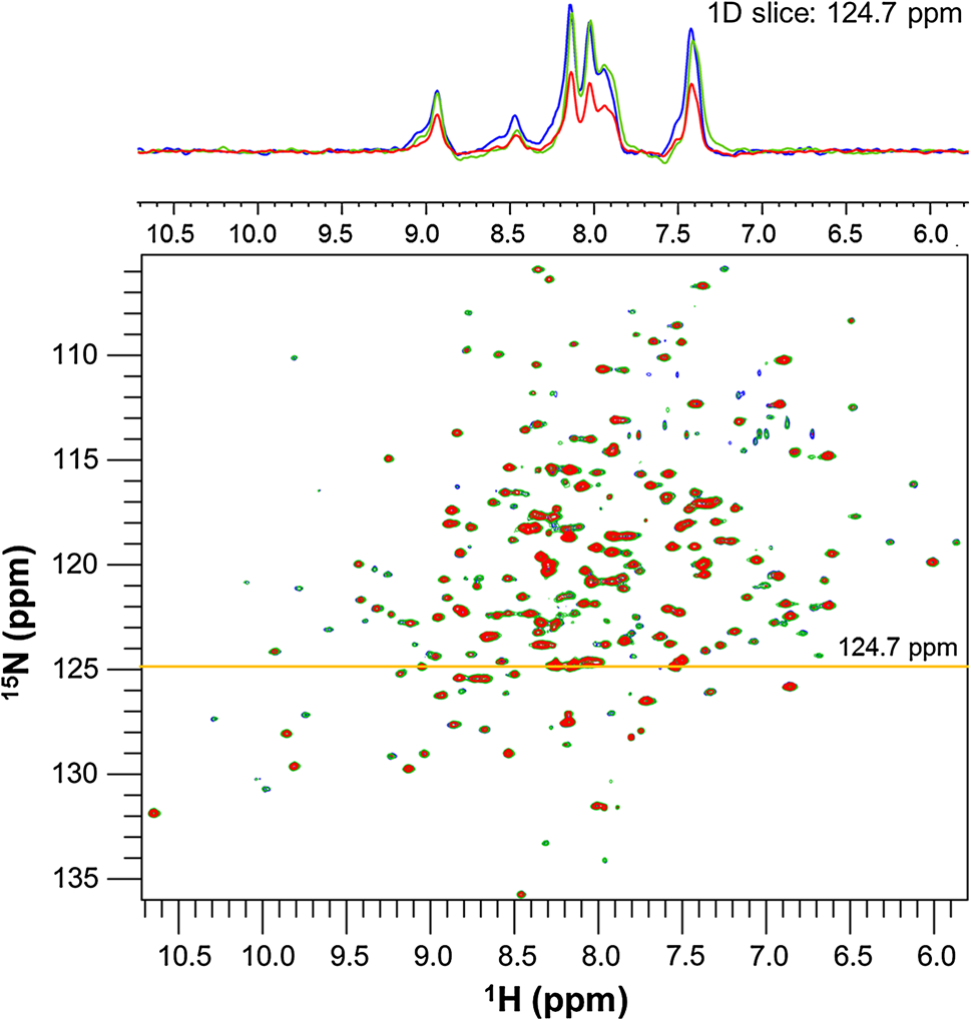
2D [^15^N,^1^H] TROSY-HSQC spectra of 400 µM (*blue*), 600 µM (*green*) or 800 µM (*red*) double labelled [^13^C,^15^N] CcP C128A with 1D overlay (*above*) showing relative ^1^H peak intensities at 124.7 ppm in the ^15^N dimension (*yellow line*). Experiments were performed at 20 °C in 20 mM NaPi, 100 mM NaCl, pH 6.0

The first evidence for weak self-association between CcP molecules appeared while optimizing the CcP concentration for the backbone amide resonance assignment of CcP (Schilder et al. 2014). Previous NMR studies on the complex between CcP and Cc were done using a 1:1 ratio at 300 µM of each protein (Bashir et al. 2010; Volkov et al. 2006, 2010b). Cc is remarkably soluble (~100 mg/mL) (Volkov et al. 2011) so the NMR sample concentration was previously limited by the solubility of CcP. However, in our hands, our CcP mutants are stable at much higher concentrations. It was hoped that indeed higher concentrations of CcP could be used during NMR experiments in order to take advantage of the increased signal intensity that would provide. To determine the optimal CcP concentration for the NMR samples, 2D [^15^N,^1^H] TROSY-HSQC spectra were obtained for samples containing 400–800 μM CcP (Fig. 2).

In a sample that does not aggregate, the signal-to-noise ratio should be proportional to the sample concentration. However, although no visible aggregation was observed in the sample, the 1D traces of the spectra showed little or no peak intensity increase when going from 400 to 600 µM and a large decrease in peak intensity when the sample concentration was further increased to 800 µM (Fig. 2). This suggests that indeed aggregation of CcP was occurring in the sample. When proteins aggregate, the intensity increase at higher concentrations is counteracted by enhanced nuclear relaxation, resulting in intensity loss due to the larger rotational correlation time of the aggregate, as compared to the monomeric state of the protein. Weak self-association has been seen in other proteins at concentrations 200–500 µM (Johansson et al. 2014; Liu et al. 2012; Tang et al. 2008a, b).

**Fig. 3:**
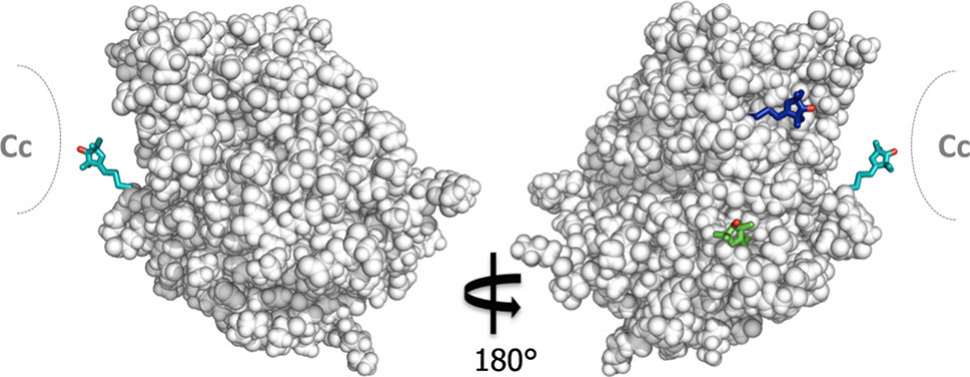
Locations of spin labels attached on the surface of CcP at positions C38 (*teal*), C200 (*blue*) and C288 (*green*) showing the nitroxide oxygen atom in *red* (PDB-entry 2PCC) (Pelletier and Kraut 1992). The binding site of Cc is shown schematically

Further evidence for a weak CcP–CcP interaction was observed during a study on the use of PRE for CcP–Cc complex structure determination (Schilder et al. 2015). We attached the spin label MTSL at three positions on CcP around the stereo-specific binding interface for Cc (Fig. 3).

Nitroxide spin labels generate measurable PRE effects up to 24 Å for a protein the size of CcP (Keizers and Ubbink 2011). Thus, no intramolecular PRE were expected beyond this limit. However, we found many PREs for residues spread across the CcP sequence, including for residues more than 24 Å from the spin label attachment site. By converting these PREs into the distances between the nitroxide oxygen atom and the amide hydrogens of CcP, the intra- and intermolecular PRE could be distinguished (Figure S1). Furthermore, upon the addition of Cc, the physiological binding partner for CcP, the suspected intermolecular PRE effects were diminished (Figure S2). The dissociation constant, *K*_D_, for the interaction between Cc and CcP is 5 μM, (Schilder et al. 2014; Volkov et al. 2009; Worrall et al. 2001) while the value for a CcP–CcP interaction would be expected to be orders of magnitude higher (Tang et al. 2008a, b; Vaynberg and Qin 2006). Therefore, assuming the CcP–CcP interaction occurs via the stereo-specific binding interface for Cc, the addition of Cc to the sample was expected to reduce the observed intermolecular PRE for CcP. This confirmed that ultra-weak intermolecular interaction occurs between CcP molecules.

**Fig. 4:**
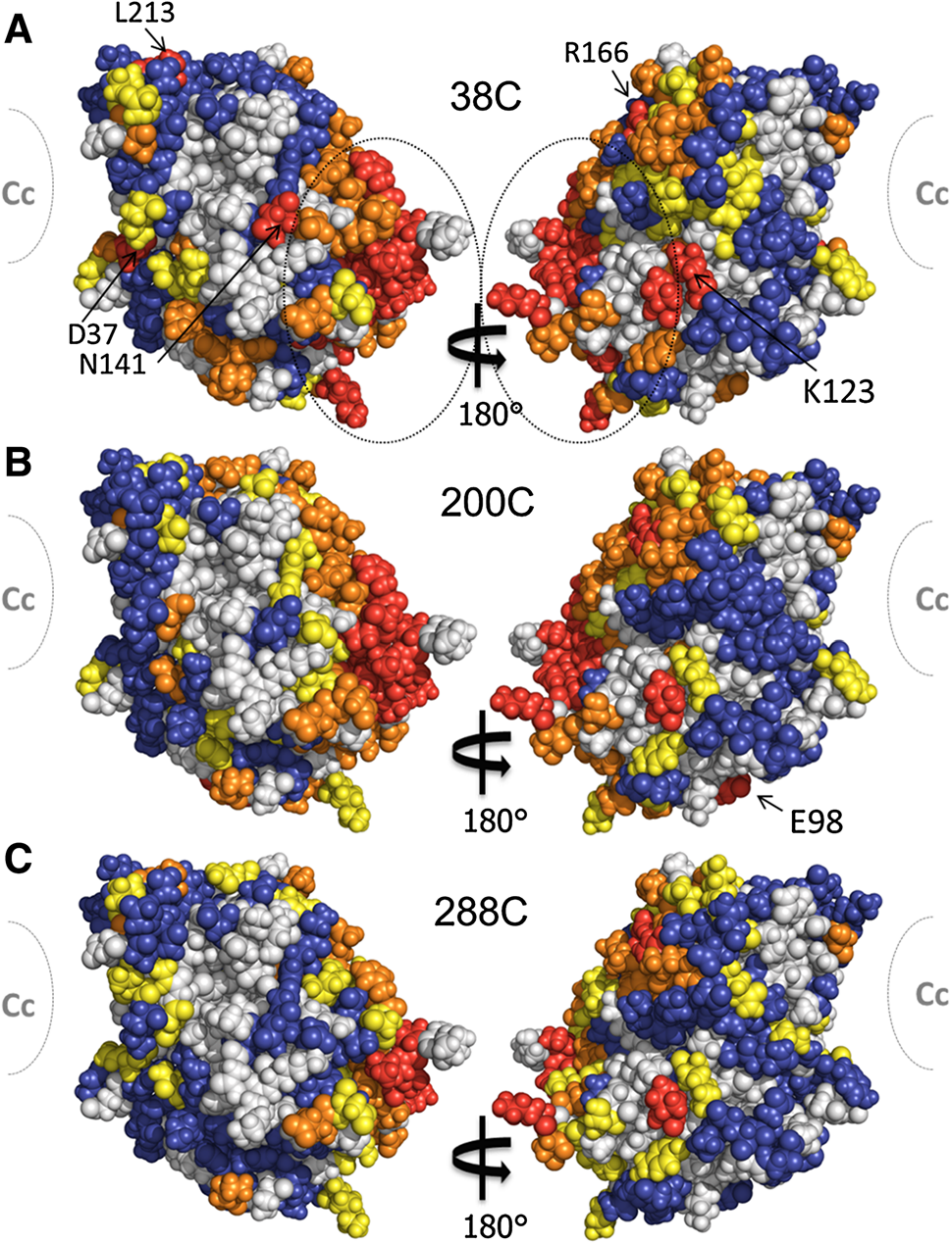
Intermolecular PRE map for CcP with MTSL at positions C38 (**a**), C200 (**b**) or C288 (**c**). The PRE effects are colour-coded on CcP (PDB-entry 2PCC) (Pelletier and Kraut 1992). The PRE effects are mostly localized around residues 3–12, 60–63, 132, 273–278, 285 (*marked with dotted oval*). The location of the stereo-specific binding site for Cc is shown schematically. Residues with R_2, para_ ≥ 100 s^−1^ are *red*, 20 s^−1^ < R_2, para_ < 100 s^−1^ are *orange*, 5 s^−1^ < R_2, para_ < 20 s^−1^ are *yellow*, R_2, para_ ≤ 5 s^−1^ are *blue* and with no data are *grey*

In order to accurately characterize this weak self-association, the PRE measurements were repeated using non-isotopically labelled CcP single mutants that were tagged with spin labels in a 1:1 mixture with WT [^2^H^15^N] isotope labelled CcP. In this way, only intermolecular PRE effects are observed, removing any possible interference from intramolecular PRE effects. The interaction between CcP molecules can then be visualized by mapping the intermolecular PRE effects on a surface model of CcP (Fig. 4).

The observed PRE effects are stronger for position C38 and C200 (Fig. 4a, b, respectively) when compared to C288 (Fig. 4c). The strength of the PRE for a given residue is dependent on the distance between that residue and the paramagnetic centre. Therefore, given the orientation of the CcP–CcP complex, spin labels at some locations may be further from the main interaction site than others, resulting in this discrepancy in PRE strength. Furthermore, as the main interaction between the CcP molecules appears to occur between the stereo-specific binding site and the backside, placement of the spin labels close to the stereo-specific binding site can interfere with the complex formation. This can result in a slightly weaker interaction for those complexes. Although the discrepancy between the PRE maps seems to be a result of the distance between the spin label attachment site and the stereo-specific binding interface (Fig. 3), the possibility of spin label interference with complex formation cannot be ruled out without further experiments.

**Fig. 5:**
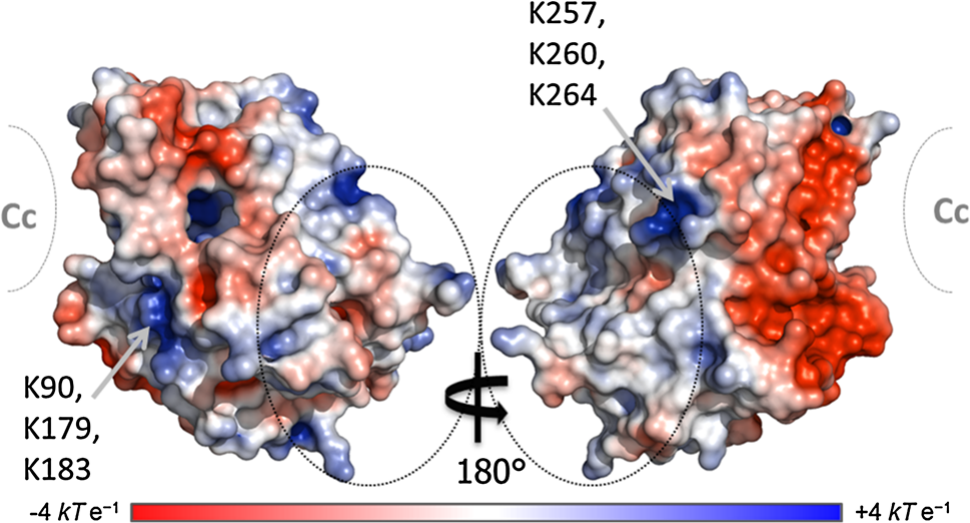
Map of the electrostatic potential generated for CcP (PDB-entry 2PCC) (Pelletier and Kraut 1992) The majority of the strong PRE effects are localized around residues 3–12, 60–63, 132, 273–278, 285 (*dotted circles*). The location of the stereo-specific binding site for Cc is marked in *grey*. The potential isocontours range from −4 *kT* e^−1^ (*red*) to +4 *kT*e^−1^ (*blue*) and were calculated using APBS (Baker et al. 2001) with an ionic strength of 120 mM at pH 6 to match experimental conditions

Several of these residues (H6, K12, H60, K123, R166, K278) are partially or fully positively charged at pH 6 and residue T3 is located beside K2, which also experienced a moderately strong PRE. Interestingly, although the majority of the remaining residues are non-polar amino acids there are also several negatively charged residues (E98, D37, D61, D132, D165). This was very unexpected for CcP, which overall is highly negatively charged (pI = 4.5), (Yonetani 1965) and particularly for a interaction involving the stereo-specific binding interface (Fig. 5).

The electrostatic potential map for CcP shows the well-known large negative patch around the stereo-specific binding interface, (Northrup et al. 1988) which has evolved to interact with the highly positively charged Cc (Volkov et al. 2011). There are also smaller negative patches on the sides and back of CcP, relative to the stereo-specific binding interface, but these are interspersed with small positive patches. These positive patches include most of the residues that experience large PRE (3–12, 60–63, 132, 273–278; dotted lines in Fig. 5) as might be expected for an interaction with the negative patch of the stereo-specific binding interface. However, the two strongest positive patches made of lysines 90, 179 and 183 and lysines 257, 260 and 264 (Fig. 5) are only moderately affected. For the first patch, residue K90 experiences moderate PRE and K183 experiences weak PRE for spin label position 38C but neither experience PRE for spin label positions 200C or 288C (no data were obtained for K179). For the second patch, although K257 experiences moderate PRE effects, K260 experiences only weak PRE for position C38 and no PRE for the other spin label positions while K264 experiences no PRE at all (Fig. 4). Therefore, although unexpected for such a highly charged protein, whose physiological interaction with Cc is driven predominantly by electrostatics, (Pelletier and Kraut 1992; Ulucan and Helms 2015) it appears that for the CcP self-association, specificity is driven by more than just electrostatics. Given the number of non-polar amino acids that also experienced strong PRE (Table 1), it appears that hydrophobic interactions are also playing a role, although no obvious hydrophobic interaction patch was identified.

**Table 1:** CcP residues strongly affected (R_2 para_ ≥ 100 s^−1^) by intermolecular PRE caused by spin labels attached at positions C38, C200 or C288

Position	CcP Residues
C38	T3, L4, V5, H6, V7, A8, V10, K12, D37, H60, N62, K123, D132, N141, D165, L213, G273, I274, T275, F276, K278, I285
C200	T3, L4, V5, H6, V7, H60, D61, N62, T63, E98, D165, R166, G189, G273, I274, T275, F276, K278, I285
C288	T3, L4, V5, D165, R166, K278, I285

The experimental PREs were measured in a sample containing 200 μL [^15^N,^2^H]-labelled CcP and 200 μL CcP tagged with MTS(L). These residues are coloured red in Fig. 4

To visualize the CcP–CcP complex, ensemble docking was employed. Modelling of weakly interacting, highly dynamic complexes generally requires an ensemble of structures to fit the observed data (Schilder and Ubbink 2013). Such an ensemble can be created by simultaneous docking of multiple copies of one of the proteins on the other driven by the experimental PREs as restraints. During the docking process experimental parameters are compared with the back-calculated ones that are averaged over all the copies of the docked proteins (Tang et al. 2006). The use of the PRE as docking restraints requires information about the fraction bound in the complex (*f*_*bound*_) as well as the rotational correlation time (τ_c_)_-_ for the complex (see “Materials and methods” section), neither of which are known. These values are linked because both are (nearly) proportional to r^6^, where r is the PRE derived distance between the spin label radical and the observed nucleus. We first estimated τ_c-_ for the whole complex to be 45 ns, which is approximately twice the value predicted for a single CcP of 20 ns (Bernado et al. 2002). Then, using this value, we estimated a fraction bound of 0.4 by establishing the lowest fraction at which the quality of fit to the experimental PREs as judged by the total docking energy no longer decreases (Figure S3). The fraction is unlikely to be larger because then larger effects on line broadening would have been expected. A fraction bound of 0.4 results in an estimated *K*_D_ of 360 μM for the CcP–CcP complex. This is approximately 70 times weaker than that of the physiological Cc–CcP complex, 5 μM, (Schilder et al. 2014; Volkov et al. 2009; Worrall et al. 2001) and is in agreement with the observation that addition of Cc to the sample diminishes the CcP–CcP interaction.

**Fig. 6:**
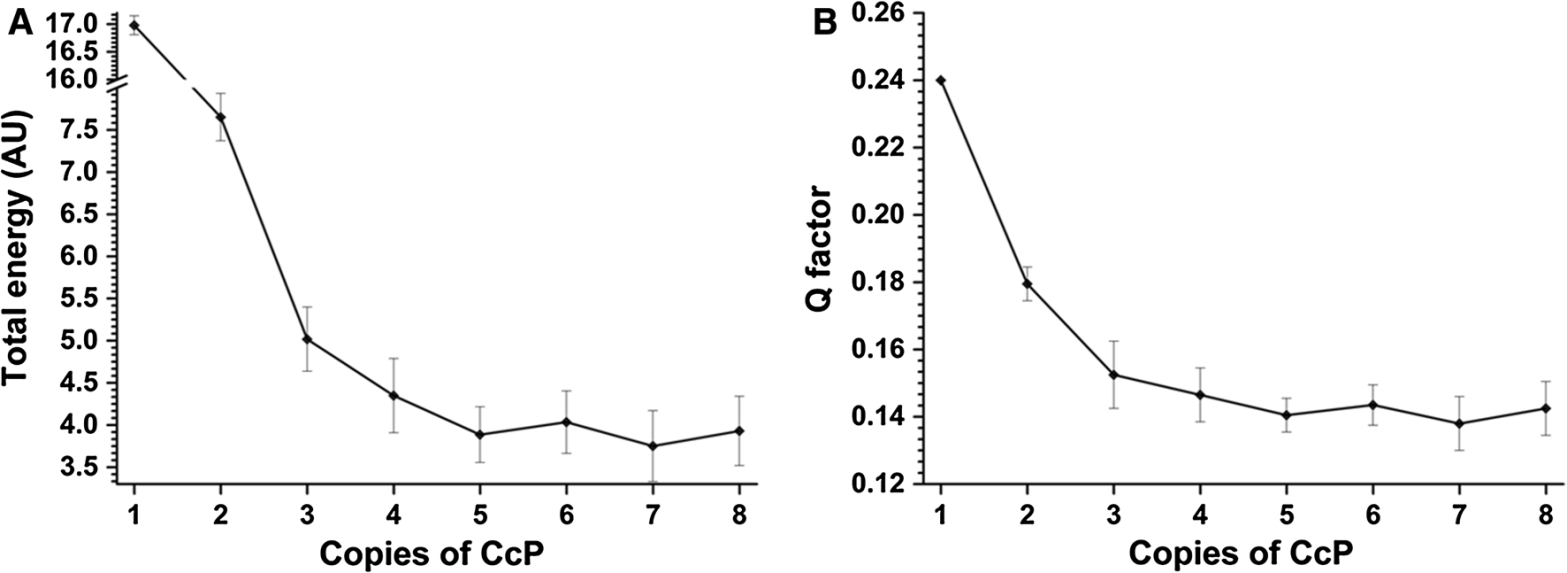
Results for docking of one or multiple copies of CcP with spin label tags on a single untagged CcP based on experimental PREs, assuming a τ_c_ of 45 ns and a fraction bound of 0.4. **a** Shows the total energy of the ensemble compared to the number of copies of CcP with spin label tags docked on a single untagged CcP and **b** the Q factor for the calculated distances between the MTSL oxygen atoms and the unlabelled CcP amide protons. The total energy is given in arbitrary units and the *error bars* represent ±1 standard deviation for the average calculated distance from the 20 lowest-energy solutions of 100 ensembles

**Fig. 7:**
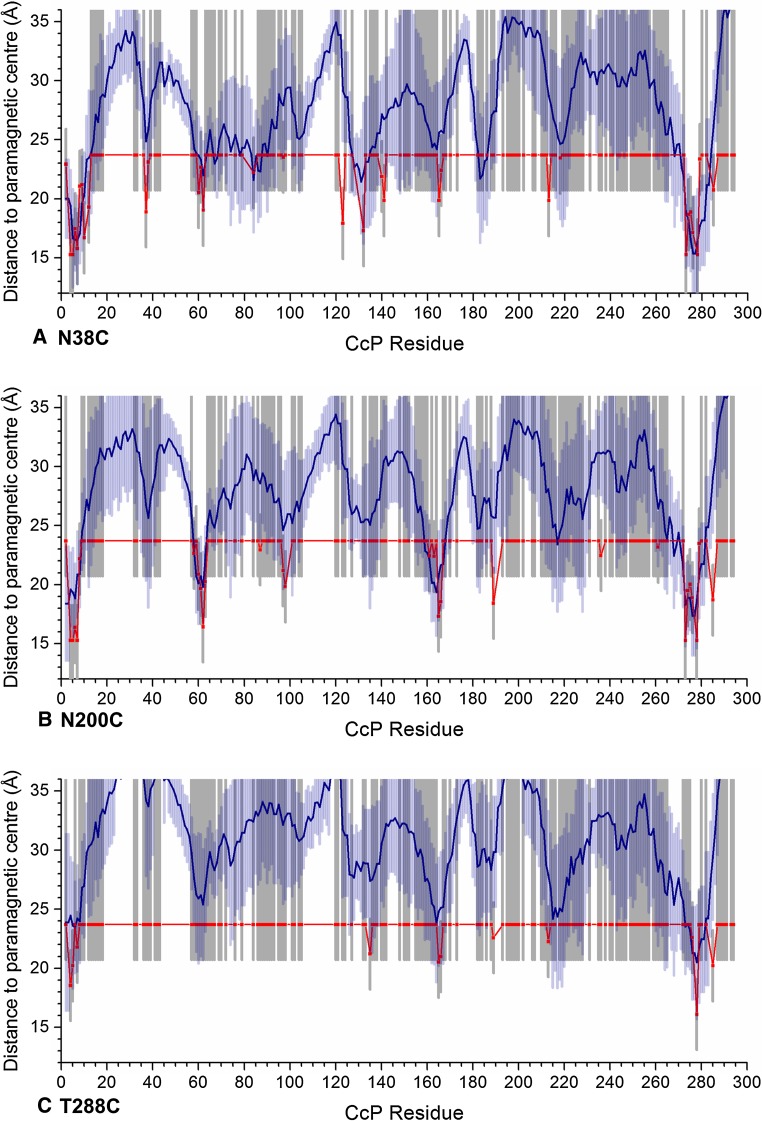
Experimental and averaged back-calculated distances between CcP C128A amide protons and the paramagnetic centre in MTSL attached to C38 (**a**), C200 (**b**) or C288 (**c**) on CcP plotted against the residue number. The *red line* represents the experimental distances with errors in *grey bars*. The averaged distances over the best 100 ensemble (n = 5) solutions from 1000 dockings are shown as a *blue line* with a spread of one standard deviation shown in *light blue bars*. The experimental data were extrapolated to 100 % bound CcP, assuming a τ_c_ of 45 ns and a fraction bound of 40 %

We found that docking five copies of spin label tagged CcP on a single copy of untagged CcP fitted the PRE data well and increasing the number of copies of spin label tagged CcP did not greatly improve the results (Fig. 6). Therefore, protein docking was done using five copies of CcP and 1000 ensembles were generated. From these, the 100 lowest energy solutions were used to back-calculate the distances between the paramagnetic centre and the CcP amide protons and compared to the experimentally observed distances (Fig. 7). The experimental and back-predicted values are shown in red and blue lines, respectively. Note that the back-predicted distances show considerable variation (light blue bars represent 1 SD from the mean). Clearly, very different sets of 5 structures can satisfy the data. When this variation is taken into account along with the experimental error margins (in grey), only a few residues show significant differences between the predicted and experimental distances. These include residues 123 and 213 for positon C38, 7, 236 and 285 for positon C200 and 4 and 189 for positon C288.

The fit between the experimental and back-predicted distances was expressed using both a Q-factor (Eq. 2) and the average violation (AV). The Q-factor can only be calculated for the class II restraints, distances between 15.3 and 23.7 Å with both an upper and lower bound (as described in “Materials and methods” section). The AV calculation can also be used for class I and III restraints, which only have an upper or lower boundary, respectively, as back-calculated distances that fall inside of those boundaries are not considered violations. The average Q factor is 0.11 and the average violation (AV) is 0.60 Å for the data of the three spin label positions. (Table 2).

**Table 2:** Q-factors and average violations (AV) for the fit of the back-calculated to the experimental distances derived from inter-molecular PRE between CcP amide protons and the paramagnetic centre in MTSL at positions C38, C200 or C288 for the best 100 ensemble (n = 5) solutions out of 1000 dockings

Position	C38	C200	C288
AV (Å)	0.76	0.68	0.35
Q-factor	0.10	0.095	0.11

**Fig. 8:**
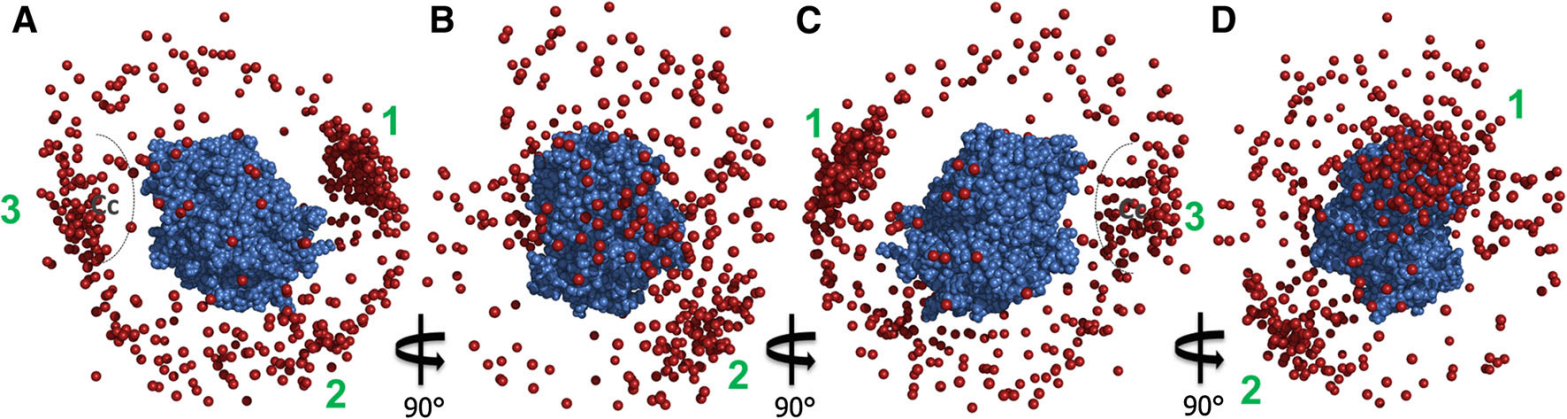
The 100 lowest-energy solutions for docking an ensemble of five copies of CcP with spin label tags on a single untagged CcP driven by intermolecular PRE data. The unlabelled CcP is shown in *blue* *spheres*. For clarity, the spin label tagged CcP copies are represented only by their haem iron atoms (*red spheres*). The numbers indicate the three main clusters of CcP positions. The stereospecific binding site for Cc is indicated schematically. The docking was done using the CcP structure taken from PDB entry 2PCC (Pelletier and Kraut 1992)

The model of the CcP–CcP complex based on the 100 ensembles of each five copies is shown in Fig. 8 as one CcP molecule in space-fill representation and the other CcP molecules shown as red spheres representing their haem irons. In line with the PRE map (Fig. 4), spin label tagged CcP samples a broad area of the untagged CcP surface. The majority of the conformations are clustered around the “back” of CcP in an area bordered by several of the residues that showed strong PRE effects (Fig. 8—cluster 1): residues 60–61, 123, 165–166, 273–278 and 285 (Table 1). At the “back” of CcP, there is also a second cluster of conformations (Fig. 8—cluster 2), bordered by several more residues that experienced strong PRE, 62-63, 132 and 141. Residues 3-12 sit between these two clusters and so likely experience effects from conformations in both clusters. From cluster 2, there is a string of low-energy conformations that extends to a cluster at the “front” of CcP (Fig. 8—cluster 3), near the stereo-specific binding site for Cc. Along this string, several more residues that experienced strong PRE are found including residues 98, 132, 37 and 213 (the latter two being located close to the stereo-specific binding site. There is also a striking absence of conformations on the “sides” of CcP (Fig. 8a, c) indicating that the interaction between CcP molecules occurs mainly between the stereo-specific binding interface and the “back” of CcP. This agrees with the PRE map (Fig. 4) and the observation that the CcP self-association can be reduced by the addition of Cc.

**Fig. 9:**
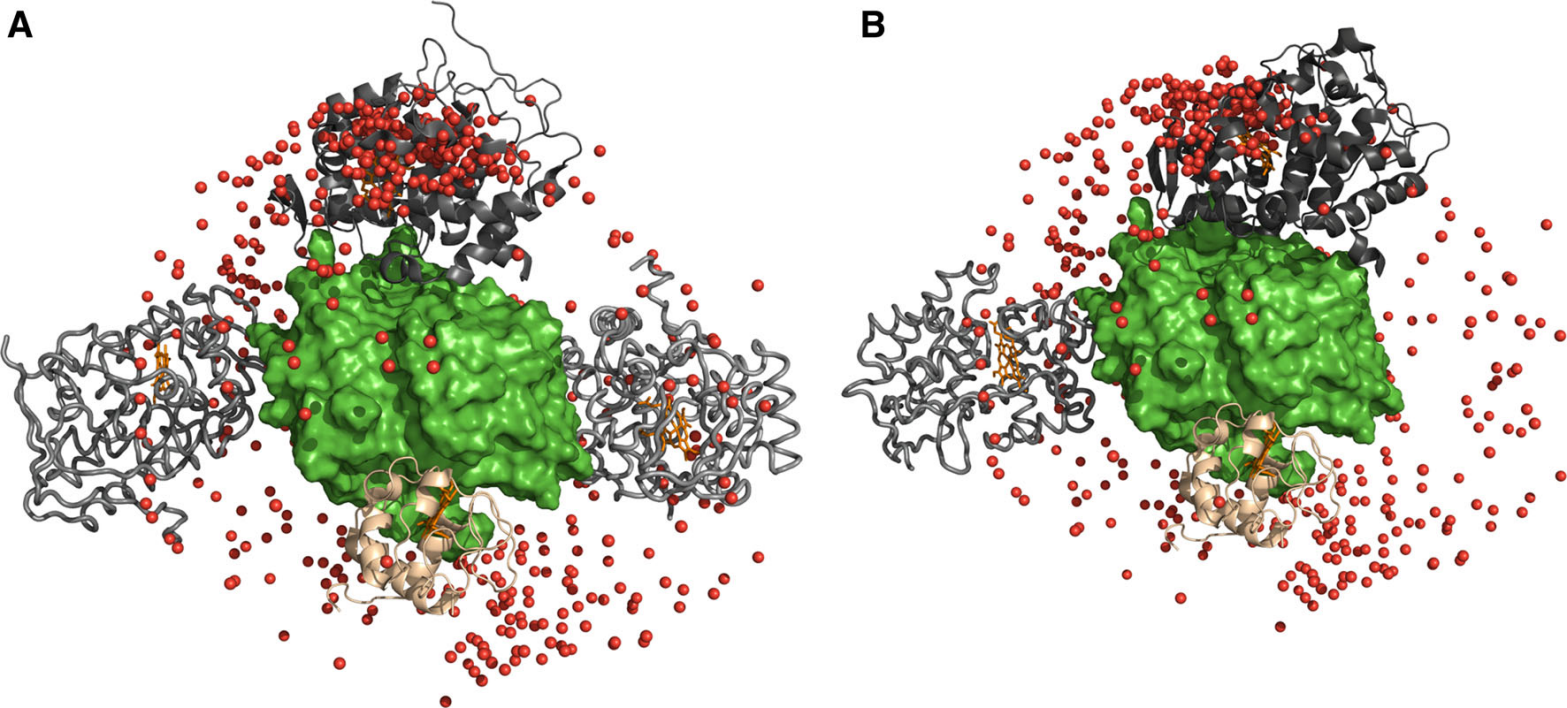
CcP crystal packing interactions. Several crystal contacts between CcP molecules in PDB entries 2PCB (Pelletier and Kraut 1992) (**a**) and 1ZBY (Bonagura et al. 2003) (**b**) are shown. The CcP (chain A) and Cc from entry 2PCC (Pelletier and Kraut 1992) are shown as *green surface* and *beige ribbon*, respectively. **a** The CcP molecules that contact the CcP chain A from 2PCC are 2PCB chain C (*grey ribbon*) and chains G and I (*grey tubes*) after alignment of 2PCB chains A, C and F, respectively, with chain A of 2PCC. **b** The CcP molecules that contact the CcP chain A from 2PCC are 1ZBY chains C (*grey tube*) and D (*grey ribbon*) after aligning 1ZBY chain B with chain A of 2PCC. The *ribbon* *and tubes* represent the CcP major and minor contact areas, respectively. Haems are shown in *orange sticks*. The ensemble of orientations obtained on the basis of PREs (shown in Fig. 8) is represented by the iron atoms of each CcP molecule, shown as *red spheres*

Interestingly, several of the strongly affected residues are located at the interface between two copies of CcP that bind head-to-tail in the X-ray crystal structure of yeast CcP with horse heart Cc, PDB entry 2PCB (Pelletier and Kraut 1992). In the structure, CcP chain C residues Q120, A193, T199, D224, G228, Y229 and E290 are all within 5 Å of one of the following residues in CcP chain A which are strongly affected in our study: T3, V5, V7, H60, T275 or K278. The orientation of chain C in 2PCB places it within cluster 1 of the CcP locations obtained by ensemble docking (Fig. 9a, grey ribbons). Two other CcP contacts involved in the crystal packing in this structure are also shown in the figure, as grey tubes. Their contact areas are much smaller, located close to isolated patches of residues showing PRE. Similarly, in the high-resolution structure of free CcP, PDB entry 1ZBY, (Bonagura et al. 2003) one large and one small CcP–CcP interaction area are observed. The larger contact places the CcP molecule within cluster 1 but in an orientation different from the one in 2PCB (Fig. 9b, grey ribbons). The smaller contact area places a CcP in a location similar to one of other two seen in 2PCB (Fig. 9b, grey tubes).

These findings suggest that the weak interactions observed in solution are also responsible for crystal packing. It has been suggested that the tight packing within the crystal lattice can mimic the crowded intracellular environment and that these interactions may be biologically relevant (Crowley et al. 2008). In this case, any potential biological relevance for the CcP dimer is unclear but it is unlikely to interfere with electron transfer from Cc due to the much greater affinity for Cc.

Finally, a note of caution should be given for the interpretation of the results from ensemble docking. Large dynamics ensembles are always under sampled by experimental data, hence the large variation in ensembles that can fit the date (Fig. 7) (Longinetti et al. 2006). Moreover, accurate conversion of the experimental PRE values into distance restraints relies on accurate values for both the τ_c_ and fraction bound, which could only be estimated. It was also assumed that the fraction bound is the same for all three spin label positions, which may not be the case if the presence of the spin label is affecting complex formation. Therefore, such models of encounter complexes should be considered only as approximations of the true encounter complex ensemble. However, they help to visualize the regions that are affected most prominently and thus very likely also responsible for the dominant interactions in the complex.

## Conclusions

In recent decades, advances in paramagnetic NMR techniques, and PRE in particular, have enabled the detailed characterization of transient, lowly populated states of weakly interacting protein complexes (Keizers and Ubbink 2011). In this study, paramagnetic NMR and the PRE effect have enabled the characterization of a weak self-association between CcP molecules and provided restraints for modelling the complex using protein docking. We show that the CcP molecules interact with each other mainly via the stereo-specific binding interface for Cc and the “backside” of the protein, as if the molecules were stacking onto each other. Such weak CcP–CcP interactions resemble those seen in CcP crystal structures and could potentially occur within the crowded intracellular environment although it is unlikely to interfere with electron transfer from Cc as the affinity between CcP and Cc is much greater than that between CcP molecules. While the biological relevance of such dimerization is not immediately obvious, the discovery of a weak CcP self-association does add to a growing body of evidence showing that many proteins exhibit a tendency for (ultra-)weak homo- or hetero- oligomerization interactions (Johansson et al. 2014; Liu et al. 2012; Tang et al. 2008a, b). The existence of a CcP–CcP interaction may also have implications for other studies on this protein, particularly when investigating weak effects, and should be taken into account when designing future experiments. Additionally, this work is a nice demonstration of the sensitivity of PRE for minor states as the level of detail the PRE data provided was much greater than that from the line broadening in the NMR spectra.

## Electronic supplementary material

Below is the link to the electronic supplementary material.

Supplementary material 1 (PDF 334 kb)
