# Supplementary material for: Weak self-association of cytochrome c peroxidase molecules observed by paramagnetic NMR
Source: J Biomol NMR. 2016 May 28;65:29–40. doi: 10.1007/s10858-016-0035-z (PMC4908164; doi:10.1007/s10858-016-0035-z)
Supplement: Supplementary file 1 — Supplementary material 1 (PDF 334 kb) [file 10858_2016_35_MOESM1_ESM.pdf]

Supplementary material for:

Weak self-association of cytochrome *c* peroxidase molecules observed by paramagnetic NMR

Jesika Schilder[1] and Marcellus Ubbink.[1]

[1]Leiden Institute of Chemistry, Leiden University, Gorlaeus Laboratories, Einsteinweg 55, 2333 CC Leiden, The Netherlands

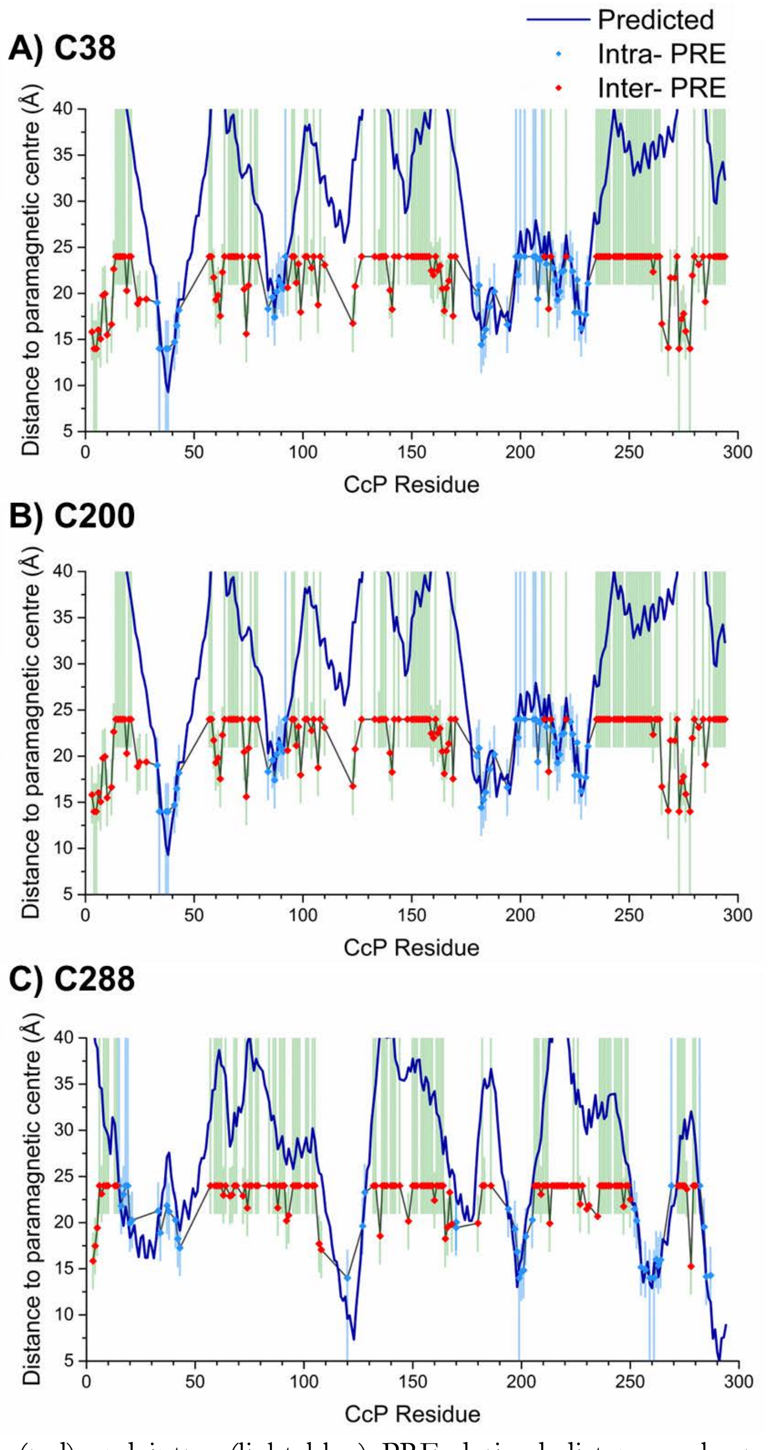

FIGURE S1 Inter- (red) and intra- (light blue) PRE derived distances observed for CcP amide hydrogen atoms generated by a nitroxide radical in MTSL attached at positions C38 **(A)**, C200 **(B)** or C288 **(C)**. The theoretical distances, back-predicted from the experimentally determined most favourable spin label orientations of a four spin label ensemble,(Schilder et al. 2015) are plotted as a dark blue line and the errors are shown in green and blue bars for the inter- and intra- PRE, respectively.

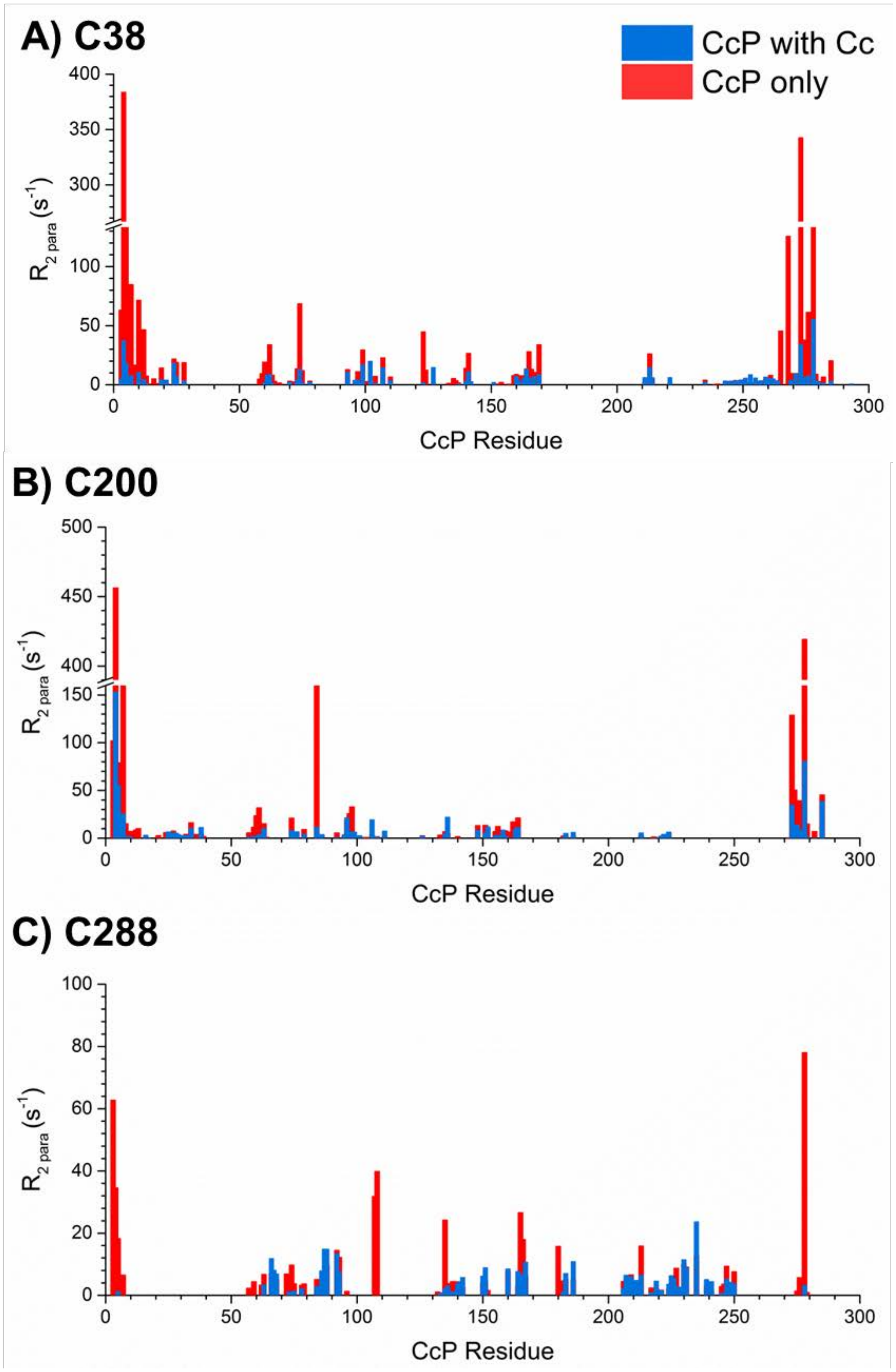

FIGURE S2 Comparison of R_{2para} for CcP amide nitrogen atoms in a sample containing only CcP (400 μL; red) or in a 1:1 with Cc (400 μL each; blue) generated by a nitroxide radical in MTSL attached to CcP at positions C38 **(A)**, C200 **(B)** or C288 **(C)**. $R_{2\ para}$ values > 130 s^{-1} cannot be measured accurately and are estimates.

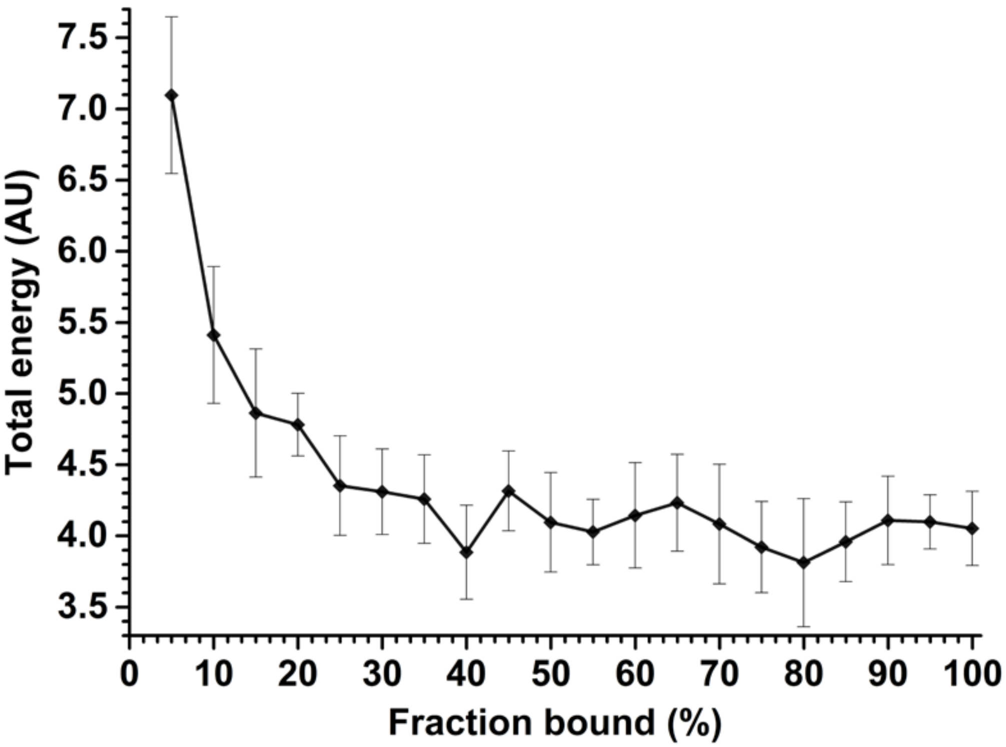

FIGURE S3 The total energy of the CcP-CcP ensemble (in arbitrary units) compared to the fraction bound for the docking of five copies of CcP with spin label tags onto a single untagged CcP based on experimental PREs assuming a τ_c of 45 ns. The error bars represent $\pm$ 1 standard deviation for the average value from the 20 lowest energy solutions of 100 ensembles.

Reference

Schilder J, Liu WD, Kumar P, Overhand M, Huber M, Ubbink M (2015) Protein docking using an ensemble of spin labels optimized by intra-molecular paramagnetic relaxation enhancement. Phys Chem Chem Phys 18:5729-5742 doi:10.1039/c5cp03781f
